# Retracted: Amodiaquine-Artesunate versus Artemether-Lumefantrine against Uncomplicated Malaria in Children Less Than 14 Years in Ngaoundere, North Cameroon: Efficacy, Safety, and Baseline Drug Resistant Mutations in *pfcrt*, *pfmdr1*, and *pfdhfr* Genes

**DOI:** 10.1155/2019/4274315

**Published:** 2019-06-20

**Authors:** Malaria Research and Treatment

Malaria Research and Treatment has retracted the article titled “Amodiaquine-Artesunate versus Artemether-Lumefantrine against Uncomplicated Malaria in Children Less Than 14 Years in Ngaoundere, North Cameroon: Efficacy, Safety, and Baseline Drug Resistant Mutations in* pfcrt, pfmdr1*, and* pfdhfr* Genes” [1]. A part of this research was done as a Fulbright Fellowship awarded to Evehe Marie Solange and performed with funding from the US National Institutes of Health (R01 AI55604) in Professor Carol Sibley's Laboratory at the Department of Genome Sciences, University of Washington Seattle, USA. Drs. Solange and Sibley did not approve publication and funding from the Fulbright Fellowship and the NIH was not acknowledged. The article lacked details of the methods and needs significant revisions.
